# Optimization, Metabolomic Analysis, Antioxidant Potential and Depigmenting Activity of Polyphenolic Compounds from Unmature Ajwa Date Seeds (*Phoenix dactylifera* L.) Using Ultrasonic-Assisted Extraction

**DOI:** 10.3390/antiox13020238

**Published:** 2024-02-15

**Authors:** Fanar Alshammari, Md Badrul Alam, Marufa Naznin, Sunghwan Kim, Sang-Han Lee

**Affiliations:** 1 Department of Food Science and Biotechnology, Graduate School, Kyungpook National University, Daegu 41566, Republic of Korea; alfnar744@gmail.com (F.A.); mbalam@knu.ac.kr (M.B.A.); 2 Food and Bio-Industry Research Institute, Inner Beauty/Antiaging Center, Kyungpook National University, Daegu 41566, Republic of Korea; 3 Department of Chemistry, Kyungpook National University, Daegu 41566, Republic of Korea; naznin@knu.ac.kr (M.N.); sunghwank@knu.ac.kr (S.K.); 4 Mass Spectroscopy Converging Research and Green-Nano Materials Research Center, Kyungpook National University, Daegu 41566, Republic of Korea

**Keywords:** Ajwa date seeds, anti-tyrosinase, hyperpigmentation, response surface methodology, artificial neural network

## Abstract

This study sought to optimize the ultrasonic-assisted extraction of polyphenolic compounds from unmature Ajwa date seeds (UMS), conduct untargeted metabolite identification and assess antioxidant and depigmenting activities. Response surface methodology (RSM) utilizing the Box–Behnken design (BBD) and artificial neural network (ANN) modeling was applied to optimize extraction conditions, including the ethanol concentration, extraction temperature and time. The determined optimal conditions comprised the ethanol concentration (62.00%), extraction time (29.00 min), and extraction temperature (50 °C). Under these conditions, UMS exhibited total phenolic content (TPC) and total flavonoid content (TFC) values of 77.52 ± 1.55 mgGAE/g and 58.85 ± 1.12 mgCE/g, respectively, with low relative standard deviation (RSD%) and relative standard error (RSE%). High-resolution mass spectrometry analysis unveiled the presence of 104 secondary metabolites in UMS, encompassing phenols, flavonoids, sesquiterpenoids, lignans and fatty acids. Furthermore, UMS demonstrated robust antioxidant activities in various cell-free antioxidant assays, implicating engagement in both hydrogen atom transfer and single electron transfer mechanisms. Additionally, UMS effectively mitigated tert-butyl hydroperoxide (t-BHP)-induced cellular reactive oxygen species (ROS) generation in a concentration-dependent manner. Crucially, UMS showcased the ability to activate mitogen-activated protein kinases (MAPKs) and suppress key proteins including tyrosinase (Tyr), tyrosinase-related protein-1 and -2 (Trp-1 and -2) and microphthalmia-associated transcription factor (MITF), which associated melanin production in MNT-1 cell. In summary, this study not only optimized the extraction process for polyphenolic compounds from UMS but also elucidated its diverse secondary metabolite profile. The observed antioxidant and depigmenting activities underscore the promising applications of UMS in skincare formulations and pharmaceutical developments.

## 1. Introduction

Extraction, the pivotal initial step in the retrieval and purification of bioactive compounds from plant sources, often relies on conventional methods characterized by lengthy extraction times and limited effectiveness [1]. To address these shortcomings, green extraction processes have been developed, offering an eco-friendly alternative. These processes are known to significantly reduce processing times, enhance heat and mass transfer rates, improve product quality, minimize solvent usage and promote the adoption of Generally Regarded as Safe (GRAS) solvents [2]. The adoption of green extraction methods not only conserves energy but also minimizes adverse impacts on the environment and human health. Among these techniques, ultrasound-assisted extraction (UAE) stands out as a green and highly efficient approach, demonstrating superior recovery yields and the preservation of target compound activities, making it particularly valuable for the extraction of antioxidants [3].

The conventional one-factor-at-a-time approach to optimization often overlooks the intricate interactions among variables, failing to guarantee optimal conditions and necessitating numerous trials, thereby increasing time, costs, and resource consumption [1]. To address this challenge, statistical methodologies such as the Box–Behnken design (BBD), a component of response surface methodology (RSM), have emerged, enabling the prediction of optimal extraction conditions and the comprehension of the relationships between extraction factors [4]. RSM encompasses a range of statistical and mathematical techniques for optimizing processes influenced by multiple variables, facilitating the development of new products and the enhancement of existing ones. It elucidates how independent variables affect processes, either individually or collectively, offering a mathematical model to represent chemical or biological procedures and assess the impact of independent factors [5]. Nonetheless, the forecast accuracy of RSM may be compromised in the presence of nonlinear relationships between variables [6]. Artificial neural networks (ANNs), with their capacity for learning algorithms and modeling nonlinear systems, are increasingly adopted as predictive tools across various disciplines, including food technology [7].

Free-radical reactions in biology play a crucial role in tissue damage and pathological events in living organisms, especially in aerobic life where lipids with polyunsaturated fatty acids are susceptible to oxidation [8]. Excess oxygen or insufficient reduction can generate reactive oxygen species (ROS) such as superoxide anions, hydroxyl radicals and hydrogen peroxide. Aerobic organisms have a natural antioxidant defense system, but if inadequate, ROSs may cause oxidative damage to macromolecules [9]. Phytochemicals with intrinsic antioxidant activity have emerged as potential remedies for oxidative stress-induced disorders. Antioxidative phenolics in plant tissues, serving various roles from structural to defensive, are believed to contribute to their medicinal actions. These compounds, studied extensively for their positive effects on human health, can orchestrate cellular protective signaling cascades, making them valuable candidates for mitigating oxidative stress-induced disorders. Exploring the therapeutic potential of these natural compounds holds promise in understanding and preventing diseases associated with free-radical reactions [8,9].

Melanogenesis, the intricate process of melanin synthesis involving melanocytes and keratinocytes, is central to skin pigmentation. Melanin, produced by melanocytes, is then transferred to adjacent keratinocytes, ultimately influencing skin color [10]. This physiological phenomenon serves multiple vital functions, including protection against harmful agents such as ultraviolet radiation (UVR) and various drugs. However, dysregulation of melanogenesis can lead to cosmetically undesirable outcomes, such as freckles, chloasma, dermatitis, and age-related skin pigmentation [10,11]. Thus, managing melanogenesis in the human epidermis is a challenging scientific and clinical pursuit. UVR exposure triggers DNA damage and activates p53, which in turn regulates tyrosinase (Tyr), tyrosinase-related protein-1 and -2 (Trp-1 and -2), through the microphthalmia-associated transcription factor (MITF) in melanocytes [12]. Additionally, several kinase proteins, including p38, c-jun N-terminal kinases (JNKs) and extracellular signal-regulated protein kinases (ERKs), influence melanogenesis [11].

The date palm, (*Phoenix dactylifera* L. Arecaceae family), is a globally popular and nutritionally significant fruit. Among its various cultivars, the Ajwa date stands out as one of the most esteemed and expensive varieties due to its ethnomedical associations with health-enhancing properties [13]. Preclinical research has highlighted its diverse health-promoting attributes, including antioxidative, anti-inflammatory, anticancer, hepatoprotective, antimicrobial, nephroprotective, antidiabetic and hyperlipidemic effects [14,15,16,17,18,19]. Additionally, the fruit is a rich source of dietary fiber, minerals, organic acids and vitamins, contributing to its nutritional and therapeutic significance, with carbohydrates constituting over 70% of its composition. Furthermore, it contains a plethora of bioactive components, such as polyphenols, encompassing phenolic acids, flavonoids and lignans [20]. Notably, the benefits of Ajwa dates extend beyond the fruit itself to its seeds, the often overlooked and underutilized byproducts of various date-related industries. These seeds, derived from technological or biological transformations of date fruits, are typically discarded or used as fertilizer or animal feed. However, they hold untapped potential as a source of high-value-added components, although limited research has explored their potential in pharmaceutical or nutraceutical product development [21].

This study unveils an innovative method to enhance the extraction of polyphenols from unmature Ajwa date seeds (UMS) by employing UAE, RSM-BBD and ANN. Through the systematic optimization of key parameters such as temperature, time and ethanol concentration, a robust model was developed to maximize polyphenol yield from UMS, representing a significant advancement in the field. Furthermore, the application of high-resolution mass spectrometry facilitated the comprehensive identification of bioactive secondary metabolites within UMS, shedding new light on their potential pharmacological benefits. Additionally, the evaluation of antioxidant potential underscores the promising applications of UMS in both food and pharmaceutical industries. An important aspect of this study is the investigation into UMS’s depigmentation properties using MNT-1 cells, accompanied by mechanistic studies. These findings not only emphasize UMS’s potential in dermatology and skincare but also lay the groundwork for future applications in this field.

## 2. Materials and Methods

### 2.1. Sample Collection and Preparation

Unmature Ajwa date fruits (Kimri stage), harvested in Al-Madina Al-Munawara, Saudi Arabia, were collected in July, 2022 and scientifically verified at the National Herbarium and Genebank of Saudi Arabia, with a voucher specimen (No. NHG005) stored for reference. UAE was conducted at a fixed 25 kHz frequency using specialized equipment (Elma Schmidbauer GmbH, Singen, Germany). Unmatured date seeds (UMS) were meticulously cleaned, air dried (40 ± 1 °C for 2 days using the laboratory dry oven) and ground (average particle size: 300 µm of diameter). Dry powder samples (1.0 g) underwent triple extraction with 10 mL of solvent, following the design in Table 1. Additionally, heat and maceration extraction followed established methods [22]. The extracted samples were filtered, concentrated in a rotary evaporator (Tokyo Rikakikai Co., Ltd., Tokyo, Japan) and lyophilized with a freeze dryer (Il-shin Biobase, Goyang, Republic of Korea). The resulting UMS extract was stored at −20 °C for subsequent experiments. The lyophilized extract was dissolved in a mixture of DMSO and dH_2_O (1:9) to create a stock solution of 100 μg/mL. Subsequently, serial dilution was performed using dH_2_O to prepare the experimental concentrations.

### 2.2. Measurement of Total Phenolic (TPC) and Total Flavonoid Content (TFC)

The TPC and TFC in UMS extracts were quantified using the Folin–Ciocalteu test and aluminum chloride colorimetric method, respectively [11]. For TPC measurement, 2 μL of a sample (100 μg/mL) was combined with 10 μL of Folin–Ciocalteu’s phenol reagent (Sigma-Aldrich, St. Louis, MO, USA). After 5 min, 100 μL of a 7% Na_2_CO_3_ solution was introduced, followed by the addition of 90 μL of dH_2_O. The mixture was then incubated in the dark for 90 min at room temperature. Absorbance was subsequently measured at 750 nm. On the other hand, for TFC measurement, 2 μL of the sample was mixed with a solution comprising 100 μL of dH_2_O, 5 μL of 5% NaNO_2_, and 10 μL of 10% AlCl_3_·6H_2_O. After 10 min, 40 μL of NaOH (1 M) was added, and the absorbance was measured against the reagent blank at 506 nm. The experiments were conducted in triplicates for each trial. Utilizing regression equations derived from calibration curves, the TPC (y = 0.0512x + 0.0018; r^2^ = 0.9835) and TFC (y = 0.014x + 0.0021; r^2^ = 0.9994) were determined. TPC was expressed as gallic acid equivalent (mg)/dry weight sample (g), and TFC as catechin equivalent (mg)/dry weight sample (g).

### 2.3. Cell-Free Antioxidant Assays

The free radical scavenging activity of UMS was evaluated using established protocols for DPPH, ABTS, superoxide, hydroxyl and nitric oxide radicals [8,9]. Percent inhibition was calculated using Equation (1) and IC_50_ values were determined for each radical to assess UMS efficacy.

(1) $$Radical\;scavenging\;activity\left(\%\;inhibition\right)=\left[\left(\frac{A-B}{A}\right)\times \;100\right]$$

where *A* and *B* denote the absorbance of the control and sample, respectively. Each sample was examined three times.

For DPPH and ABTS radical scavenging assays, various concentrations of the sample (2 μL) were mixed with 198 μL of DPPH (0.2 M in 50% methanol) and ABTS solutions (2.5 mM potassium persulfate and 7 mM ABTS). The mixtures stood for 10 min at room temperature in the dark, and absorbance readings were taken at 517 nm and 734 nm for the DPPH and ABTS assays, respectively. Ascorbic acid served as the reference antioxidant.

Additionally, DPPH and ABTS scavenging was expressed as µmol ascorbic acid equivalents per gram (μmol AAE/g) of UMS using calibrated regression equations (DPPH: y = 0.0069x + 0.035; r^2^ = 0.9905 and ABTS: y = 0.0083x + 0.0002; r^2^ = 0.9989).

Superoxide radical generation was verified using the non-enzymatic PMS/NADH complex, where NBT is reduced to formazan. Samples (2 µL) were mixed with a superoxide radical generation mixture (73 µM NADH, 50 µM NBT and 15 µM PMS in PBS), incubated for 30 min, and absorbance was read at 562 nm [9]. Gallic acid served as the reference antioxidant.

For hydroxyl radical scavenging activity, samples (5 µL) were added to the Fenton reaction mixture (3.6 mM deoxyribose, 0.1 mM EDTA, 0.1 mM ascorbic acid, 1 mM H_2_O_2_ and 0.1 mM FeCl_3_ in PBS). After a 1 h incubation at 37 °C, 1% TBA and 2.8% TCA were added, heated, and absorbance was measured at 532 nm. Quercetin was the standard.

For nitric oxide measurement, samples (10 µL) were mixed with sodium nitroprusside (10 mM) in PBS, incubated for 150 min, then reacted with Griss reagent (Sigma-Aldrich, St. Louis, MO, USA). Absorbance was measured at 546 nm using catechin as the reference compound.

Moreover, the reducing power potential of UMS was evaluated through cupric-reducing antioxidant capacity (CUPRAC) and ferric-reducing antioxidant power (FRAP) assays, following Alam et al. [8]. In FRAP and CUPRAC assays, samples (2 µL) were mixed with an FRAP reagent (20 mM FeCl_3_ and 10 mM TPTZ in acetic acid buffer), and absorbance was measured at 595 nm. For CUPRAC, samples were mixed with a solution of CuCl_2_, neocuproine (Sigma-Aldrich, St. Louis, MO, USA) and ammonium acetate buffer, incubated for 1 h at 25 °C, and absorbance was measured at 450 nm. The obtained values were expressed as (mmol AAE/g) of UMS using standard curves for each assay (CUPRAC: y = 0.0065x + 0.039; r^2^ = 0.9975; and FRAP: y = 0.013x + 0.0465; r^2^ = 0.9889).

### 2.4. Cell Culture and Intracellular ROS Generation Assay

RAW 264.7 macrophages (ATCC, Rockville, MD, USA) were maintained in DMEM supplemented with 10% FBS and 100 µg/mL each of streptomycin and penicillin, under standard culture conditions (37 °C, and 5% CO_2_). Cells (5 × 10^5^/mL) were seeded in 96-well plates and incubated for 12 h. Subsequently, they were treated with UMS (6.25–100 µg/mL) for 24 h, both alone and in combination with t-BHP (oxidative stress inducer). Cellular toxicity was assessed using the MTT assay, while reactive oxygen species (ROS) generation induced by t-BHP was evaluated by the DCFH-DA method, as previously described [8].

### 2.5. Effect of UMS on Melanin Content

Cells (5 × 10^5^ cells/mL) were cultured in a 24-well plate (BD Falcon, Bedford, MA, USA) overnight. Subsequent to media replacement, cells were exposed to UMS (25–100 μg/mL) or arbutin (100 μg/mL). Post-3-days, PBS-washed cells were lysed with 1 N NaOH, and absorbance at 405 nm was measured using a microplate reader (Thermo Fisher Scientific, Vantaa, Finland). The percentage of melanin inhibition was calculated using Equation (2).

(2) $$Melanin\;production\left(\%\;inhibition\right)=\left[\left(\frac{A-B}{A}\right)\times \;100\right]$$

where *A* and *B* represent the absorbance of non-treated cells and treated with UMS or arbutin (positive control), respectively [11].

### 2.6. Preparation of Cell Lysates and Western Blotting

Cell lysates were treated with sodium dodecyl sulfate (SDS) buffer (3M, Maplewood, MN, USA) and denatured at 100 °C for 5 min. Proteins (30 µg) were separated on a 10% SDS-polyacrylamide gel, transferred to nitrocellulose membranes (Whatman, Dassel, Germany) and blocked with 5% skim milk in TBST buffer. After blocking, membranes were probed with primary antibodies (Supplementary Table S1), followed by secondary antibodies (anti-rabbit IgG-HRP; BioWorld Technology, St. Louis Park, MN, USA). Antigen–antibody reactions were detected using an ECL solution system (Perkin Elmer, Waltham, MA, USA) [11].

### 2.7. Single-Factor Experiment

Polyphenolic compound extraction was studied through single-factor experiments, varying the ultrasonic time (10–50 min), temperature (30–70 °C) and ethanol concentration (25–90%). Optimal ultrasonic-assisted extraction conditions were then determined based on these results (Table S2).

### 2.8. Experimental Design of RSM for the Extraction Process

In this study, BBD was employed to optimize the extraction process of UMS for maximizing TPC and TFC. The independent extraction variables considered were ethanol concentration (X_1_), extraction time (X_2_) and temperature (X_3_), while the response variables of interest were TPC (Y_1_) and TFC (Y_2_). The relationships between these variables were modeled using a second-order polynomial Equation (3):

(3) $$Y=\beta_{0}+\sum_{i=1}^{n}\beta_{i}X_{i}+\sum_{i=1}^{n}\beta_{ii}X_{ii}^{2}+\sum_{i}^{n-1}\sum_{j}^{n}\beta_{ij}X_{ij}$$

where *Y* represents the response variable; *X_i_* and *X_j_* are the coded independent variables; *β*_0_ is the constant coefficient; and *β_i_*, *β_ii_* and *β_ij_* are the coefficients for linear, quadratic and interaction effects, respectively. The outcomes of these interactions were visualized through three-dimensional (3D) surface plots.

### 2.9. Artificial Neural Network (ANN) Modeling

A multilayer perceptron (MLP) neural network was used to establish a link between independent variables (X_1_, X_2_ and X_3_) and response variables Y_1_ and Y_2_ using a backpropagation feed-forward ANN model [23]. The dataset was divided into training (70%), validation (15%) and testing (15%) sets. Training was conducted using a hit and trial technique to minimize the mean square error (MSE) calculated from Equation (4). Two different types of neural networks, feed-forward and cascade feed-forward, were utilized, employing the Broyden–Fletcher–Goldfarb–Shanno (BFGS) and Levenberg–Marquardt backpropagation (trainlm) algorithms. The ANN model’s architecture consisted of three layers, as depicted in Supplementary Figure S1, with the output generated through nonlinear activation functions in the hidden layer. Several statistical parameters, including *R*^2^, *RMSE*, *AAD* and *SEP*, were computed using specific Equations (5)–(8), to evaluate and compare the predictive performance of the ANN and RSM. This approach allowed for a robust assessment of the nonlinear relationships between input and output variables.

(4) $$MSE=\frac{1}{N}\sum_{i=1}^{N}(Y_{ANN}-Y_{Exp}{)}^{2}$$

(5) $$R^{2}=1-\frac{\sum_{i=1}^{n}(x_{i}-x_{ik}{)}^{2}}{\sum_{i=1}^{n}(x_{ik}-x_{z}{)}^{2}}$$

(6) $$RMSE=\sqrt{\frac{1}{n}\sum_{i=1}^{n}(x_{i}-x_{ik}{)}^{2}}$$

(7) $$ADD\;\%=\left[\frac{\sum_{i=1}^{n}\left(\left|x_{ik}-x_{i}\right|/x_{ik}\right)}{n}\right]\times 100$$

(8) $$SEP\;\%=\frac{RMSE}{y_{m}}\times 100$$

where *Y_p_* is the predicted response; *Y_e_* is the observed response; *Y_m_* is the average response variable; *n* is the number of experiments

### 2.10. Validation of the Model

To ascertain the optimal extraction parameters for UMS, a combination of response surface and Derringer’s desirability function was employed. Each response was transformed into a unique desirability function, ranging from 0 to 1 based on their relative desirability, from lowest to highest. These component functions were then integrated into a total desirability function using Equation (9) [1].

(9) $$D={\left(d_{1}^{w1}d_{2}^{w2}\ldots .d_{n}^{wn}\right)}^{1/\sum wi}$$

To assess the agreement between observed and expected UMS outcomes, calculations of the RSD and RSE were performed using Equations (10) and (11), respectively. According to the criteria set forth, data were considered consistent with predictions when RSD and RSE values fell below 10% and 5%, respectively [24,25].

(10) $$RSD\left(\%\right)=\frac{Standard\;deviation\;between\;predicted\;and\;actual\;values}{Mean\;values\;between\;predicted\;and\;actual\;values}\times \;100$$

(11) $$RSE(\%)=\frac{\left(Actual\;value–Predicted\;value\right)}{Predicted\;value}\times 100$$

### 2.11. Analysis of Chemical Compounds by ESI–MS/MS

In this study, negative-mode ESI–MS experiments were conducted using the Q-Exactive™ Orbitrap Mass Spectrometer (Thermo Fisher Scientific Inc., San Jose, CA, USA). Sample infusion into the ESI source was accomplished directly at a rate of 20 µL/min utilizing a 500-µL syringe (Hamilton Company Inc., based in Reno, NV, USA), coupled with a syringe pump (Harvard in Holliston, MA, USA, model 11). Data acquisition was conducted through Xcalibur 3.1 with Foundation 3.1. (San Jose, CA, USA). The ESI–MS conditions featured a mass resolution of 140,000, a mass spectra range of *m*/*z* 50–1000, sheath gas at a flow rate of 5 and auxiliary gas at 0. Additionally, a spray voltage of 4.20 kV, capillary temperature at 320 °C and automatic gain control set at 5 E 6 were applied. MS/MS studies employed three stepwise normalized collision energies (10, 20 and 30) [9]. The identification of *m*/*z* peaks was achieved by comparing calculated (exact) masses of deprotonated (M–H) adducts with *m*/*z* values and ESI–MS/MS fragmentation patterns from in-house and online databases such as FooDB [26], METLIN [27] and CFM-ID 4.0 [28]. The chemical structure was drawn using ChemDraw Professional 15.0 (PerkinElmer, Waltham, MA, USA).

### 2.12. Statistical Analysis

Experimental data underwent robust analysis employing Design Expert 11 (Stat-Ease, Minneapolis, MN, USA) and GraphPad Prism 9 (GraphPad Software 9.0.2, San Diego, CA, USA). MATLAB’s Neural Network Toolbox^TM^ (MATLAB R2020a, MathWorks, Natick, MA, USA) facilitated artificial neural network (ANN) analysis. Results, presented as mean ± standard deviation from a minimum of three independent experiments (*n* = 3), were rigorously scrutinized for statistical significance at *p* < 0.001, < 0.01 and < 0.05. Design Expert 11 enabled RSM analysis. GraphPad Prism 9 conducted one-way analysis of variance and Tukey’s multiple comparison test for all biological activities, deeming *p* < 0.05 statistically significant.

## 3. Results and Discussion

### 3.1. Single Factor Analysis

In this study, experiments were conducted to assess the individual effects of three key factors—ethanol concentration, extraction time and temperature—on the extraction yields of TPC and TFC from UMS.

**Figure 1:**
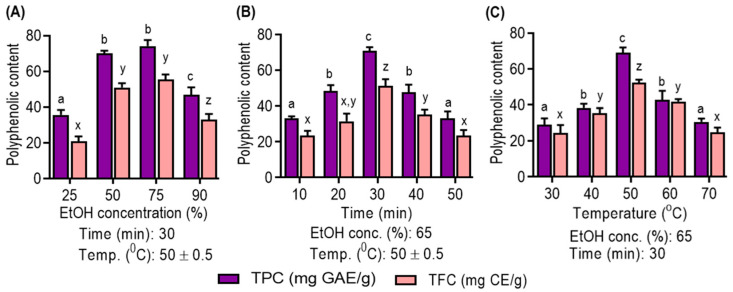
Effect of (**A**) ethanol concentration; (**B**) extraction time; and (**C**) extraction temperature on UMS extraction on ethanol concentration, time and temperature single factor on the yield of TPC and TFC of UMS extract. Different letters represent statistical significance (*p* < 0.05) between each group. (a, b, c and d for TPC; x, y and z for TFC).

As shown in Figure 1A, the choice of solvent concentration is pivotal. Ethanol, particularly at 75% concentration, is demonstrated to be the optimal choice due to its compatibility with the solubility of polyphenolic compounds. Notably, aqueous ethanol is favored for its low toxicity and cost-effectiveness, which enhance its efficiency in polyphenolic extraction, as corroborated by recent studies [4]. Figure 1B highlights the impact of the ultrasonic extraction time. Extended times (10–30 min) are beneficial for increased polyphenolic yield, but prolonged extraction negatively affects yields. This decline is attributed to structural degradation and solvent loss through vaporization, diminishing mass transfer efficiency [29]. Figure 1C emphasizes the role of temperature in polyphenolic compound extraction. Elevated temperatures (30 °C to 50 °C) enhance yields by disrupting cellular structures and reducing viscosity, consistent with prior research [30]. However, temperatures above 50 °C result in reduced yields due to decreased acoustic cavitation intensity, diminished extraction efficiency and potential thermosensitive compound degradation [31].

### 3.2. Fitting of the RSM and ANN Models

Table 1 presents the outcomes of 17 extraction scenarios, summarizing the experimental parameters and results. The yields of TPC and TFC in UMS extracts exhibited a range from 60.23 ± 0.79 to 76.65 ± 0.49 mgGAE/g and 37.62 ± 1.10 to 59.40 ± 0.89 mgCE/g, respectively. The peak TPC and TFC values were achieved at the central point of the design (X_1_: 60% EtOH; X_2_: 30 min; and X_3_: 50 °C). Both RSM and ANN predictions were closely aligned with experimental results, with minimal discrepancies. Quadratic polynomial equations (Equations (12) and (13)) were utilized to model the response variables (TPC and TFC, respectively), accounting for their variation concerning the extraction factors.

(12) $$TPC\;\left(Y_{1}\right)=75.77+1.21X_{1}-0.9912X_{2}+0.1438X_{3}-8.12X_{1}^{2}-5.59X_{2}^{2}-5.73X_{3}^{2}-0.6275X_{1}X_{2}+0.5975X_{1}X_{3}+0.5100X_{2}X_{3}$$

(13) $$TFC\;\left(Y_{2}\right)=57.42+1.44X_{1}+0.6375X_{2}+0.3691X_{3}-10.86X_{1}^{2}-6.81X_{2}^{2}-6.83X_{3}^{2}-0.1651X_{1}X_{2}+1.50X_{1}X_{3}+0.5177X_{2}X_{3}$$

As described in Table 2, ANOVA was utilized to evaluate the statistical significance of second-order quadratic model equations. Significance levels were determined by *p*-values, classifying terms as significant (*p* < 0.05), highly significant (*p* < 0.01) or exceptionally significant (*p* < 0.001). Conversely, terms with *p*-values above 0.05 were considered nonsignificant.

The statistical significance of the model equations, as determined by the F-test and *p*-values, underscores their reliability. The high F-values (for 107.80 TPC and 46.54 for TFC) with *p*-values less than 0.0001 confirm the models’ significance. Furthermore, lack of fit tests yielded non-significant *p*-values (for 0.1772 TPC and 0.3516 for TFC), affirming the appropriateness of the second-order polynomial models for predicting total polyphenolic extraction yields [1]. The determination coefficient (R^2^) demonstrates the model’s adequacy, with values of 0.9928 for TPC and 0.9836 for TFC, indicating that over 99% of the variation in total polyphenolic yield can be explained by the model. High adjusted (R^2^ adj: 0.9836 and 0.9624 for TPC and TFC, respectively) and predicted determination coefficients (R^2^ pred: 0.9192 and 0.8503 for TPC and TFC, respectively) further confirm the correlation between the observed and predicted data. Furthermore, low coefficient of variation (C.V.) values (1.21 for TPC and 3.40 for TFC) and high Adeq. precision values (25.0494 for TPC and 16.9654 for TFC) suggest a high degree of precision and reliability in the experimental data.

**Figure 2:**
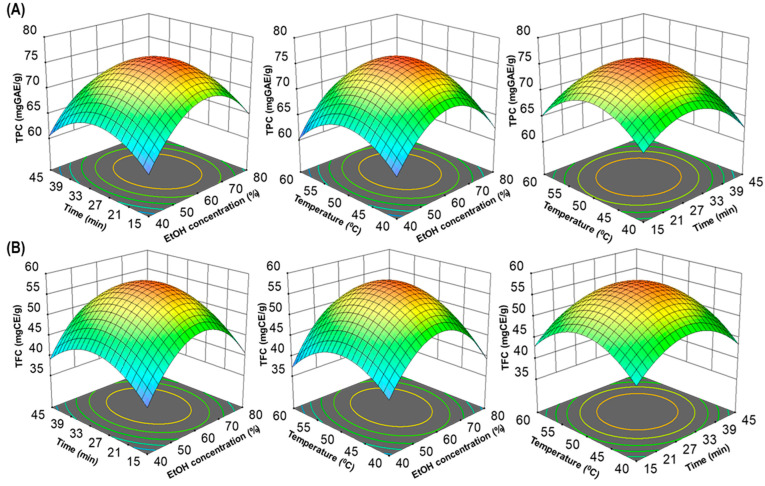
The three-dimensional (3D) response surface plots of UMS extraction on ethanol concentration, time and temperature for TPC (**A**) and TFC (**B**).

In addition, to visualize interactions between independent variables, 3D surfaces and contour plots were generated using multiple linear regression equations. These graphical representations (Figure 2A,B) help elucidate the main and cross-product effects of independent variables on the response variables, enhancing the understanding of the underlying processes. This comprehensive statistical analysis supports the robustness and validity of the model for predicting total polyphenolic extraction yields. The three-dimensional response surface analysis in RSM examined the relationship between TPC, TFC and extraction parameters. Contour plot shapes conveyed the significance of mutual interactions. Elliptical contours implied negligible interactions, while circular contours indicated significant interactions [22,25]. In Figure 2A,B, all response surfaces were convex, affirming the appropriate selection of variable ranges for ultrasonic time, temperature and ethanol concentration, highlighting their collective impact on TPC and TFC extraction yields.

There is a growing body of evidence supporting the superiority and sophistication of artificial neural network (ANN) modeling over RSM, making ANNs a promising alternative for intricate nonlinear multivariate modeling in various fields, particularly in biological processes. ANNs are inspired by the human CNS and its intricate network of interconnected neurons, enabling complex computations in response to input data [32,33]. In this study, experimental data were subjected to ANN modeling for validation. The predicted values from the ANN model closely matched the observed values (Table 1), affirming the model’s appropriateness.

**Figure 3:**
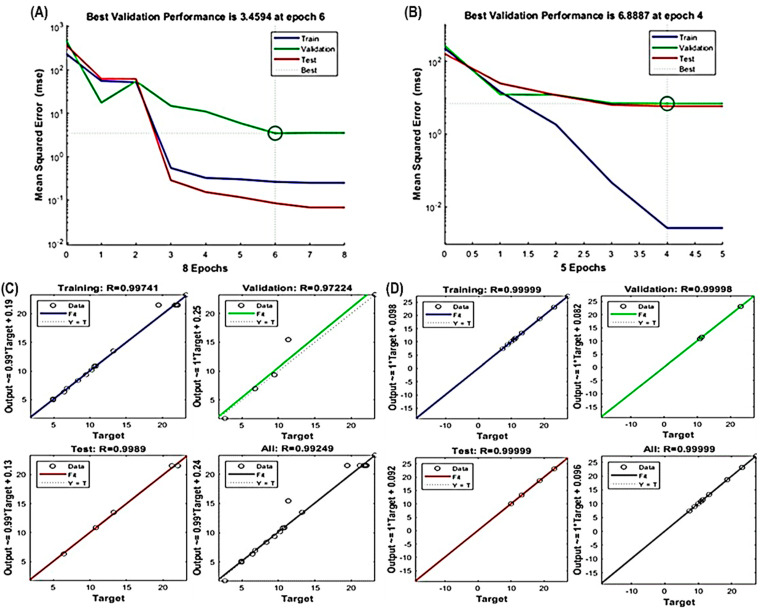
Evaluation of ANN model performance. Assessing the validation of the constructed ANN model for (**A**) TPC and (**B**) TFC. Illustration of the optimal multilayer perceptron (MLP) architecture used in training, testing and validating the regression analysis to minimize errors for ANN model development for (**C**) TPC and (**D**) TFC.

The ANN model effectively captured the nonlinear relationships between extraction parameters (X_1_, X_2_ and X_3_) and response variables (Y_1_ and Y_2_), as evidenced in Figure 3A–D. Like RSM, the fitness and significance of the ANN model relied on various parameters, including R^2^ values for training, validation, testing, overall error reduction and the number of epochs to prevent overfitting or underfitting. Notably, the best validation performance for TPC occurred at epoch 6, and for TFC at epoch 4 (Figure 3A,B). Furthermore, high R^2^ values exceeding 0.97224 for TPC and 0.99998 for TFC (Figure 3C,D) underscore the model’s precision and its potential to accurately represent the complex relationships between the variables.

### 3.3. Comparison of the Prediction Abilities of the RSM and ANN Models

The comparative evaluation of RSM and ANN models for predicting TPC and TFC in UMS revealed the superior performance of the ANN model (Table 3). With higher R^2^ values (0.9963 for TPC and 0.9912 for TFC) than RSM (0.9928 for TPC and 0.9836 for TFC), the ANN model exhibited an enhanced predictive capability. Lower AAD and RMSE values indicated a better fit. The ANN model also exhibited lower SEP values (0.0171 for TPC and 0.0326 for TFC), further emphasizing its accuracy. The ANN model’s flexibility in approximating nonlinear systems surpassed the RSM model, being constrained to second-order polynomial regression. Additionally, the ANN model’s efficiency in handling multiple responses in a single run outperformed the RSM model, often requiring multiple runs for multi-response optimization. Dadgar et al. emphasized the ANN model’s superiority in accuracy, precision and fitting experimental data to target responses, establishing it as a more effective tool in this context [34].

### 3.4. Model Validation and Comparison with Other Extraction Methods

**Figure 4:**
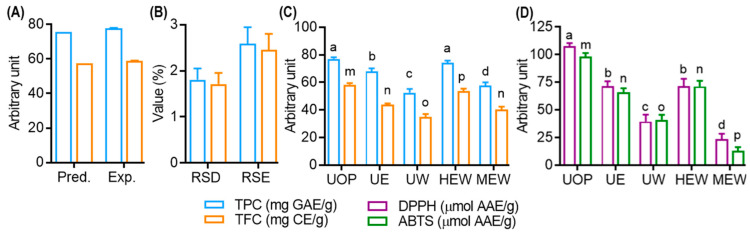
Model validation and comparative study of TPC and TFC of various extraction methods. (**A**) TPC and TFC value of optimized condition. (**B**) Relative standard deviation (RSD) and relative standard error (RSE) value of optimized condition. (**C**) Effect of various extraction techniques on the yield of TPC and TFC of UMS extract. (**D**) Effect of various extraction techniques on the DPPH and ABTS radical scavenging effects of UMS extract. Different letters represent statistical significance (*p* < 0.05) between each group. (a, b, c and d for TPC; m, n, o, and p for TFC). U: ultrasonic assisted extraction; H: heat assisted extraction; M: maceration extract; OP: optimized condition; E: 100% EtOH; W: 100% aqueous; EW: 75% EtOH; AAE: ascorbic acid equivalent.

The simultaneous optimization of TPC and TFC in UMS extracts was achieved using Derringer’s desirability function [34]. The maximum overall desirability (D = 0.953) was attained under specific conditions: ethanol concentration (X_1_, 61.43 ≅ 61.00%), extraction time (X_2_: 29.60 ≅ 30.00 min) and extraction temperature (X_3_, 50.24 ≅ 50.00 °C). The contour plot as a function of ethanol concentration, extraction time and temperature at the optimum condition are presented in the Supplementary Materials, Figure S2A–C. As described in Figure 4A,B, the predictive model was validated with triplicate experiments, resulting in TPC and TFC values of 77.52 ± 1.55 mgGAE/g and 58.85 ± 1.12 mgCAE/g, respectively, with low RSD% (1.80 for TPC and 1.71 for TFC) and RSE% (2.59 for TPC and 2.45 for TFC).

A comparative study was performed to evaluate the efficiency of the optimized extract (UOP) obtained through UAE in comparison to traditional methods. As shown in Figure 4C, UOP exhibited significantly higher TFC values compared to other extracts. Remarkably, UOP showed no significant difference in TPC compared to HEW (head assisted extraction coupled with 75% EtOH), while surpassing other methods. Similarly, UOP demonstrated superior 2,2-diphenyl-1-picrylhydrazyl (DPPH) and ABTS radical scavenging activity compared to all other extracts, while UE and HEW displayed no significant difference in their scavenging capacities (Figure 4D). These findings underscore the remarkable efficiency of UAE, which not only improves polyphenol yield and antioxidant activity but also minimizes extraction time. This improved efficiency may be attributed to various factors affecting ultrasonic energy transmission, including gas diffusion, gas–liquid phase transitions and chemical reactions [29].

### 3.5. Identification of Secondary Metabolites in UMS by High-Resolution Mass Spectroscopy

In the optimized UMS extracts, secondary metabolites were analyzed using ESI–MS/MS in the negative ionization mode. A total of 104 compounds were successfully identified, relying on the precursor ion mass, characteristic fragmentation patterns and comparisons with the reference standards, literature and online databases (Table 4). The significance of these findings was assessed based on confidence levels: Level 1 for compounds confirmed with reference standards, Level 2 for probable identifications supported by MS^n^ data from the literature, and Level 3 for tentative candidates [35].

In Table 4, compounds **1**–**13** were identified as phenolic compounds in UMS based on mass fragmentation patterns. Notably, the phenolic acids in UMS were often in glucosidic form or conjugated with quinic and shikimic acid. While compounds **1**–**12** had been reported in various date cultivars in previous studies [1,17,22,24], compound **13,** with a monoisotopic mass [M–H]^−^ of *m*/*z* 747.1895 and the molecular formula C_38_H_36_O_16_, was newly identified as 3-*O*-feruloyl-7-*O*-acyl-feruloyl-4-*O*-caffeoyl-quinic acid in the Ajwa date.

Flavonoids are a diverse group of polyphenolic compounds found in plants, categorized into seven subclasses based on their structural variations: flavonols, flavones, isoflavones, anthocyanidins, flavanones, flavanols and chalcones [36]. Among the flavonoids identified, flavone aglycones, including apigenin (**14**)**,** luteolin (**15**)**,** chrysoeriol (**17**) and methoxysinensetin (**21**), were identified along with their glycosides (compounds **25**, **34**, **46**, **47** and **54**) and sulfate conjugates as luteolin hexosyl sulfate (**41**) and chrysoeriol hexosyl sulfate (**42**) [17,20,22,24]. Quercetin (**18**), a major flavonol aglycone, was found alongside numerous flavonol glycosides (**29**, **30**, **35**, **38**, **40**, **43**, **48**, **49**, **52** and **55–57**), which were prevalent across various date variants [20,22,24]. Furthermore, UMS unveiled novel flavonol glycoside conjugates with hydroxycinnamic acids, specifically kaempferol 3-(3″,6″-di-*p*-coumaroylgalactoside) **(51),** quercetin 3-(6′′′′-*p*-coumaryl sophorotrioside) (**58**) and quercetin 3-(6″-caffeoyl sophorotrioside) (**59**), as determined through MS^n^ data and previous studies [37,38]. Moreover, UMS contained flavonoid diversity, with compounds **23** and **26** identified as naringenin glycosides (flavanones) and compounds **28**, **33** and **39** characterized as biochanin A glycoside, afrormosin glycoside and luteone glycoside (isoflavones). Compounds **16**, **22**, **27**, **31**, **32** and **36** were confirmed to be flavanols, mainly catechin/epicatechin and their glycosidic and gallate conjugates [24]. Interestingly, epigallocatechin (**20**) and epigallocatechin caffeate (**37**) were discovered for the first time in UMS. This study also revealed the presence of proanthocyanidins in UMS, including procyanidin A2 (**44**), procyanidin B2 (**45**), procyanidin B2-gallate (**50**) and epicatechin-(4beta->8)-epigallocatechin 3′-gallate (**53**), marking the first-time identification of these proanthocyanidins in UMS.

Terpenes, characterized by their five-carbon isoprene units, represent a significant category of secondary metabolites. Terpenoids, on the other hand, are derivatives of terpenes, displaying a variety of functional groups and methyl group rearrangements. Terpenoids are categorized into monoterpenes, sesquiterpenes, diterpenes, sesterpenes and triterpenes based on their carbon unit composition. Mass spectrometry of terpenoids unveils distinct ions resulting from the loss of neutral molecules, such as CH_3_ (15 Da) H_2_O (18 Da), CO (28 Da), COO (44 Da) and CH_3_COOH (60 Da). Furthermore, pseudo-molecular ions undergo retro-Diels–Alder (RDA) fragmentation reactions. Terpenoid glycosides can also generate terpenoid aglycones by shedding sugar units [39,40,41,42]. Comparing fragmentation patterns to the prior literature, sesquiterpenoids (compounds **60**–**65**, **68** and **72**) and their lactone derivatives, including absindiol **(66)** and cymaroside A (**75**), were identified. Compound **74**, with a deprotonated ion [M–H]^−^ at *m*/*z* 427.1974 and molecular formula C_21_H_32_O_9_, was recognized as the sesquiterpene glycoside cichorioside M. Additionally, two monoterpenoids (**67** and **71**), two diterpenoids (**69** and **70**) and two triterpenoids (**73** and **76**) were characterized in the study [40,42,43,44]. Notably, the study marks the first-ever report of the presence of terpenoids in UMS.

Compounds **77**–**79** have been unequivocally identified as lignan glycosides. Notably, compound **77**, 1,2-di-(syringoyl)-hexoside, had been previously discovered in Ajwa date pulp [22]. Compounds **78** and **79**, newly discovered in UMS, exhibited deprotonated ions [M–H]^−^ at *m*/*z* 567.2084 and 581.2236, respectively and generated base peaks at *m*/*z* 405.15 and 419.17 by losing the glycosyl moiety (162 Da), confirming their identities as citrusin B (**78**) and lyoniresinol glucoside (**79**) [45,46].

Mass spectrometric analysis and data from the literature and online databases aided in the identification of compounds 80–86 as carboxylic acids and compounds 87–100 as fatty acids (Table 4). These findings are consistent with previous research [20,26,27,28,47,48,49]. Compounds **102** and **103**, identified as N-acetyl-α-neuraminic acid and 1-deoxynojirimycin hexoside, were previously reported in Ajwa date pulp [24]. In contrast, compounds **101** and **104**, dihydrojasmonic acid and icariside D1, were newly found in UMS, characterized by their deprotonated ions [M–H]^−^ at *m*/*z* 211.1335 and 415.1609, respectively, which was supported by their mass fragmentation behavior documented in earlier studies [50].

### 3.6. Antioxidant Effect of UMS

**Figure 5:**
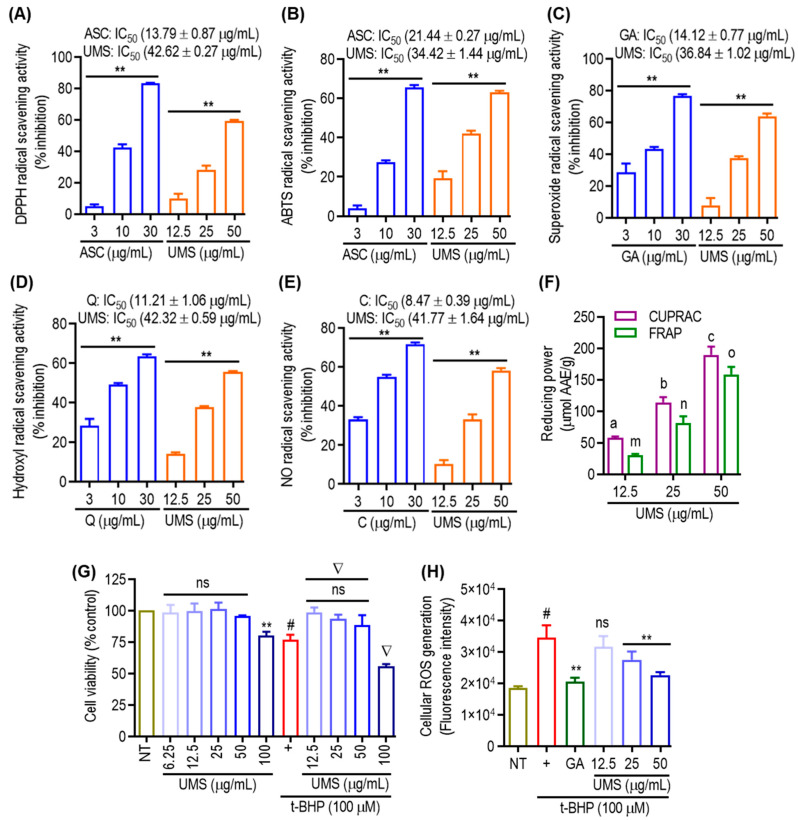
Antioxidant effects of UMS. Cell-free (**A**) DPPH; (**B**) ABTS; (**C**) superoxide; (**D**) hydroxyl; and (**E**) NO radical scavenging activities of UMS. ** p < 0.01 vs. NT. (**F**) Reducing power activities of UMS in CUPRAC and FRAP assay. Different letters represent statistical significance (*p* < 0.05) between each group. (a, b and c for CUPRAC; m, n and o for FRAP). (**G**) Cell viability of UMS in RAW 264.7 cells. ** p < 0.05 vs. NT, ^#^ *p* < 0.01 vs. NT, ns: non-significant vs. NT, ^∇^ *p* < 0.01 vs. UVB alone. (**H**) Effect of UMS on t-BHP induced cellular ROS generation. ^#^ *p* < 0.001 vs. NT, ** p < 0.01 vs. UVB alone, ns: non-significant vs. UVB alone. ASC: ascorbic acid; GA: gallic acid; Q: quercetin and C: catechin. AAE: ascorbic acid equivalent.

The assessment of antioxidant activities in phytochemicals necessitates a comprehensive understanding of various molecular mechanisms. In this investigation, multiple methodologies were employed to evaluate the antioxidant potential of UMS. The DPPH, ABTS, superoxide, hydroxyl and NO radical scavenging assays, as well as CUPRAC and FRAP assays, were utilized. As depicted in Figure 5A,B, UMS demonstrated a dose-dependent and significant DPPH and ABTS radical scavenging potential, exhibiting IC_50_ values of 42.62 ± 0.27 μg/mL and 34.42 ± 1.44 μg/mL, respectively. In comparison, the positive control, ascorbic acid, exhibited an IC_50_ value of 13.79 ± 0.87 μg/mL and 21.44 ± 0.27 μg/mL in the DPPH and ABTS assays, respectively, indicating that UMS not only engage in hydrogen atom transfer but also operates through a single electron transfer mechanism. The evaluation of superoxide and hydroxyl radical scavenging abilities employed the PMS–NADH superoxide-generating system and Fenton reaction, respectively [8]. Figure 5C,D illustrate UMS’s robust potential to scavenge superoxide and hydroxyl radicals, with IC_50_ values of 36.84 ± 1.02 μg/mL and 42.32 ± 0.59 μg/mL, respectively. In contrast, the positive controls, gallic acid and quercetin, had IC_50_ values of 14.12 ± 0.77 μg/mL and 11.21 ± 1.06 μg/mL for superoxide and hydroxyl radical scavenging, respectively. These results suggest that UMS employ a single electron transfer mechanism for its antioxidant activity. Moreover, UMS exhibited dose-dependent NO radical scavenging activity with an IC_50_ value of 41.77 ± 1.64 μg/mL, while the positive control catechin had an IC_50_ value of 8.47 ± 0.39 μg/mL (Figure 5E). Furthermore, CUPRAC and FRAP assays were performed, revealing UMS’s notable reduction capability with values of 189.42 ± 12.98 and 158.32 ± 12.05 μmol AAE/g at 50 μg/mL, respectively (Figure 5F).

Furthermore, t-BHP, a well-known short-chain lipid peroxide analogue that is frequently used to produce oxidative stress in cells, was employed to examine cellular responses to oxidative stress in both cellular and tissue settings in order to evaluate the potential of UMS in attenuating cellular oxidative stress. The induction of oxidative stress by t-BHP resulted in notable cell death. However, pretreatment with UMS and gallic acid effectively mitigated cellular toxicity at non-toxic concentrations (Figure 5G). Additionally, as depicted in Figure 5H, UMS demonstrated a dose-dependent reduction in the generation of cellular ROSs, further highlighting its antioxidative properties.

This study corroborates existing evidence indicating that date seed extracts, particularly from Ajwa seeds, exhibit high radical scavenging activity in various cell-free antioxidant methods. Ajwa seeds surpass date flesh in antioxidant properties, positioning them as a promising natural source of antioxidants. The attributed effectiveness of Ajwa seeds in addressing various conditions is likely linked to polyphenolic compounds functioning as reducing agents, free radical scavengers and hydrogen donors. The antioxidant properties and polyphenolic composition of date seeds are influenced by factors such as genetic diversity, soil conditions, maturity stages, storage conditions and extraction methods. Furthermore, UMS, containing cinnamic acid, benzoic acid hydroxylated derivatives and various flavonoids, is implicated in its antioxidant properties, offering protection against oxidative-stress-induced diseases such as inflammation, hyperlipidemia and diabetes.

### 3.7. Depigmenting Effect of UMS on Hyperpigmented Melanocyte (MNT-1) Cells

Melanogenesis, the process of melanin production, plays a crucial role in skin pigmentation and can be implicated in various skin conditions [12]. Mushroom tyrosinase is a well-established enzyme used to test compounds that inhibit melanogenesis [51] and the results indicated that UMS significantly suppressed mushroom tyrosinase activity in a concentration-dependent manner, with a lower IC_50_ value (48.60 ± 1.02 μg/mL) compared to the positive control, arbutin (IC_50_ = 131.03 ± 2.01 μg/mL) (Figure S4).

**Figure 6:**
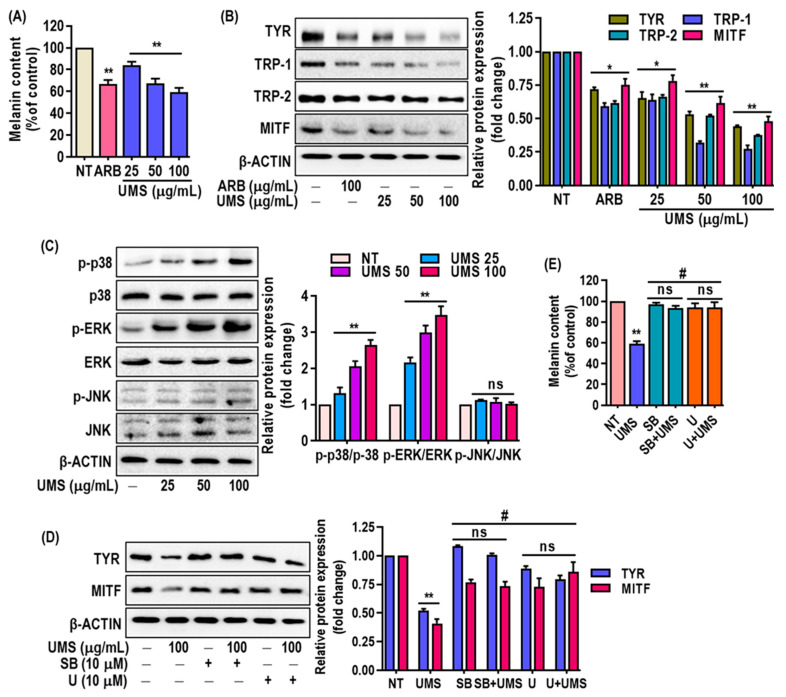
Effects of UMS on melanogenesis in MNT-1 cells. Cells were cultured with UMS (25–100 μg/mL) for 3 days. (**A**) Melanin content was determined. Western blot analysis of (**B**) melanogenesis factors such as Tyr, Trp-1, -2 and MITF, (**C**) MAPK proteins including p38, ERK and JNK. MNT-1 cells were co-treated with UMS and selective inhibitors of ERK (U0126) and p38 (SB239063). (**D**) MITF and Tyr levels were determined by western blot analysis, and (**E**) melanin content was also determined. * *p* < 0.05, ** *p* < 0.01 vs. NT, ^#^ *p* < 0.01 vs. UMS alone, ns: non-significant vs. NT, ARB: arbutin.

Furthermore, the study examined the impact of UMS extract on melanin levels in MNT-1 cells and revealed a dose-dependent reduction (Figure 6A) without causing cytotoxicity (Figure S5). This reduction in melanin content was associated with the downregulation of key melanogenesis-related proteins, including Tyr, Trp-1, -2 and MITF, as confirmed through western blot analysis (Figure 6B). The inhibition of MITF expression is particularly significant, as MITF is a master regulator of melanogenesis. The presence of flavonoids and procyanidins in the UMS extract aligns with the observed depigmenting effects. Flavonoids (luteolin, taxifolin, quercetin catechin, epigallocatehin, procyanidinA2 and B2) have been previously linked to anti-melanogenic activity in the cosmetic industry [52,53], and the mass spectrometric analysis confirmed the abundance of these compounds in the UMS extract. Moreover, the study delved into the underlying mechanisms of UMS’s depigmenting effects. The study demonstrated that the UMS extract stimulated the p38 and ERK signaling pathways in MNT-1 cells, which play roles in regulating melanogenesis. In contrast, UMS fail to trigger JNK activation in MNT-1 cells (Figure 6C). Moreover, in order to confirm the involvement of the p38 and ERK signaling pathways in mediating UMS’s depigmenting effects, specific inhibitors for p38 and ERK were administered either alone or in combination with UMS. The findings indicated that inhibiting p38 and ERK selectively successfully reversed the depigmenting effects of UMS. This association linked the activation of these pathways to the reduction of MITF expression and the inhibition of Tyr expression (Figure 6D,E). This supports earlier research that demonstrated that polyphenolic-rich plant extracts achieve depigmentation through similar MAPKs’ signaling-mediated MITF downregulation [11,54].

## 4. Conclusions

This pioneering study investigated the optimization of UAE conditions for extracting bioactive compounds from unmature Ajwa date seeds using both RSM and ANN modeling. High-resolution mass spectrometry analysis identified phenolic acids, flavonoids, lignans and fatty acids in the extract. The ANN model outperformed the RSM model, exhibiting higher accuracy and reliability, with the optimal conditions determined to be 61% ethanol, 29 min of extraction time and an extraction temperature of 50 °C, while the extract/solvent ratio was fixed to 1:10 (g/mL). Under these conditions, the extract yielded maximum TPC of 77.52 ± 1.55 mg GAE/g and TFC of 58.85 ± 1.12 mg CE/g. Furthermore, UMS showed a potent antioxidant activity in various cell-free antioxidant assays and the mitigation of t-BHP induced cellular ROS generation. The date industry generates thousands of discarded byproducts, such as date pomace and seeds, that are rich with bioactive compounds. New aspects of using these byproducts to produce high-nutritional-value food products have recently attracted interest. Additionally, this study highlighted the extract’s potential as an anti-melanogenic agent, showing its ability to inhibit mushroom tyrosinase, reduce melanin levels and modulate melanogenesis-related proteins. The activation of p38 and ERK signaling pathways further supports its potential for pigmentation-related skin care products. This research underscores the significance of Ajwa date seeds as a source of bioactive compounds and encourages its further exploration in dermatology and cosmetics, including isolating bioactive markers and in vivo testing for therapeutic applications.

## Figures and Tables

**Table 1:** Box–Behnken design (BBD) for the independent variables and corresponding response value (experimental).

Run	Independent Variables			Response					
	EC (%) (X_1_)	Time (min) (X_2_)	Temp (°C) (X_3_)	TPC (mg GAE/g) (Y_1_)			TFC (mg CE/g) (Y_2_)		
				RSM (prd.)	ANN (prd.)	Exp.	RSM (prd.)	ANN (prd.)	Exp.
1	80	15	50	64.99	65.59	65.15 ± 1.15	40.71	41.66	39.25 ± 1.05
2	80	30	40	62.30	62.60	61.56 ± 0.52	39.28	39.25	40.25 ± 0.98
3	60	45	60	64.13	64.89	63.55 ± 1.15	45.29	44.57	44.80 ± 0.56
4	60	30	50	75.70	76.88	75.26 ± 1.01	57.41	57.95	58.32 ± 0.28
5	60	30	50	75.77	76.85	75.56 ± 0.89	57.41	57.20	57.01 ± 1.15
6	60	30	50	75.87	75.32	76.15 ± 0.69	57.41	57.69	59.40 ± 0.89
7	80	45	50	61.57	61.60	61.95 ± 1.00	41.65	41.25	41.53 ± 0.79
8	60	15	60	65.62	66.29	64.55 ± 1.15	42.98	42.77	43.83 ± 0.69
9	60	45	40	62.97	62.57	63.35 ± 0.69	43.52	44.09	42.67 ± 1.09
10	40	15	50	61.21	61.55	60.92 ± 0.59	37.50	38.83	37.62 ± 1.10
11	60	15	40	65.80	66.33	66.39 ± 1.01	43.28	42.62	43.77 ± 0.99
12	40	30	60	60.34	60.95	61.09 ± 1.15	37.14	36.99	36.17 ± 1.09
13	40	45	50	60.38	61.75	60.23 ± 0.79	39.10	39.56	40.56 ± 1.00
14	60	30	50	75.17	76.60	76.65 ± 0.49	57.41	58.25	56.32 ± 1.02
15	60	30	50	75.77	76.05	75.25 ± 0.89	57.41	57.85	56.01 ± 0.58
16	40	30	40	61.10	61.59	60.89 ± 0.92	39.41	40.66	38.79 ± 0.65
17	80	30	60	64.03	65.60	64.15 ± 1.09	43.03	44.25	43.64 ± 0.45

X_1_: Ethanol concentration (EC); X_2_: time; X_3_: temperature; TPC: total phenolic content; TFC: total flavonoid content; RSM (prd.): predicted value by response surface method; ANN (prd.): predicted value by artificial neural network method; mgGAE/g: mg gallic acid equivalent/g dry weight of sample; mgCE/g: mg catechin equivalent/g dry weight of sample.

**Table 2:** ANOVA for quadratic model (function: none).

ANOVA for Quadratic Model for TPC							
Source	RC	SS	DF	MS	F-Value	*p*-Value	
Model		632.77	9	70.31	107.80	<0.0001	Significant
Intercept	75.77						
Linear terms							
X_1_	1.21	11.71	1	11.71	17.96	0.0039	Significant
X_2_	−0.9912	7.86	1	7.86	12.05	0.0104	Significant
X_3_	0.1438	0.1653	1	0.1653	0.2535	0.6301	Not Significant
Interaction terms							
X_1_X_2_	−0.6275	1.58	1	1.58	2.41	0.1641	Not significant
X_1_X_3_	0.5975	1.43	1	1.43	2.19	0.1825	Not significant
X_2_X_3_	0.5100	1.04	1	1.04	1.60	0.2470	Not Significant
Quadratic terms							
X_1_^2^	−8.12	277.93	1	277.93	426.15	<0.0001	Significant
X_2_^2^	−5.59	131.43	1	131.43	201.52	<0.0001	Significant
X_3_^2^	−5.73	138.10	1	138.10	211.75	<0.0001	Significant
Lack of Fit		3.07	3	1.02	2.74	0.1772	Not significant
Pure error		1.49	4	0.3733			
R^2^							0.9928
Adjusted R^2^							0.9836
Pred. R^2^							0.9192
Adeq Precision							25.0494
C.V. %							1.21
**ANOVA for quadratic model for TFC**							
**Source**	**RC**	**SS**	**DF**	**MS**	**F-value**	***p*-value**	
Model		1016.65	9	112.96	46.54	<0.0001	Significant
Intercept	57.42						
Linear terms							
X_1_	1.44	16.62	1	16.62	6.85	0.0346	Significant
X_2_	0.6375	3.25	1	3.25	1.34	0.2851	Not Significant
X_3_	0.3691	1.09	1	1.09	0.4489	0.5243	Not Significant
Interaction terms							
X_1_X_2_	−0.1651	0.1090	1	0.1090	0.0449	0.8382	Not significant
X_1_X_3_	1.50	9.04	1	9.04	3.72	0.0949	Not significant
X_2_X_3_	0.5177	1.07	1	1.07	0.4416	0.5276	Not Significant
Quadratic terms							
X_1_^2^	−10.86	496.93	1	496.93	204.73	<0.0001	Significant
X_2_^2^	−6.81	195.12	1	195.12	80.39	<0.0001	Significant
X_3_^2^	−6.83	196.58	1	196.58	80.99	<0.0001	Significant
Lack of Fit		8.88	3	2.96	1.46	0.3516	Not significant
Pure error		8.11	4	2.03			
R^2^							0.9836
Adjusted R^2^							0.9624
Pred. R^2^							0.8503
Adeq Precision							16.9654
C.V. %							3.40

X_1_: ethanol concentration (%); X_2_: time (min); X_3_: temperature (°C). RC: Regression coefficient; SS: sum of squares; DF: the total degrees of freedom; MS: mean square.

**Table 3:** Comparison of the prediction ability of RSM and ANN.

Parameters	TPC		TFC	
	RSM	ANN	RSM	ANN
R^2^	0.9928	0.9963	0.9836	0.9912
RMSE	6.7139	1.7760	3.8464	2.2384
AAD (%)	0.9237	0.1390	0.7828	0.2201
SEP (%)	0.0649	0.0171	0.0561	0.0326

R^2^: correlation coefficients; RMSE: root mean square error; AAD: absolute average deviation; SEP: standard error of prediction.

**Table 4:** List of possible identified compounds of UMS using ESI–MS/MS in the negative ionization mode ([M–H]^−^).

Groups	No.	Compound Name	EF	OM (*m*/*z*)	CM (*m*/*z*)	MS/MS	CL
Phenolic acids and derivatives	1	*p*-Coumaroyl aspartic acid	C_13_H_13_NO_6_	278.0669	278.0664	260.05, 234.07, 216.06	2
	2	4-Hydroxybenzoyl glucose	C_13_H_16_O_8_	299.0773	299.0766	137.02, 163.02	1
	3	Coumaroylshikimic acid	C_16_H_16_O_7_	319.0824	319.0817	173.04, 163.03, 145.02	2
	4	Vanillic acid glucoside	C_14_H_18_O_9_	329.0879	329.0872	167.03, 152.02, 123.04	1
	5	Caffeoyl shikimic acid	C_16_H_16_O_8_	335.0771	335.0772	179.01, 161.03, 155.03, 137.05	1
	6	Glucosyringic acid	C_15_H_20_O_10_	359.0985	359.0978	197.04, 182.01, 153.05	2
	7	Caffeic acid derivatives	C_18_H_18_O_9_	377.0853	377.0878	341.10, 215.03, 179.06, 161.04	2
	8	Sinapic acid hexoside	C_17_H_22_O_10_	385.1154	385.1135	223.06, 205.05	1
	9	Sinapoylspermine	C_21_H_36_N_4_O_4_	407.2649	407.2658	350.20, 279.13, 201.20	2
	10	Methyl 4,6-di-*O*-galloyl-glucose	C_21_H_22_O_14_	497.0927	497.0931	345.05, 183.12, 169.05, 125.01	2
	11	Caffeoyl shikimic acid hexoside	C_22_H_26_O_13_	497.1278	497.1295	335.01, 178.02, 135.02	2
	12	Cinnamoyl-1,2-digalloyl glucose	C_29_H_26_O_15_	613.1126	613.1193	483.07, 443.09, 169.01, 147.04	2
	13	3-*O*-feruloyl-7-*O*-acyl-feruloyl-4-*O*-caffeoyl-quinic acid	C_38_H_36_O_16_	747.1895	747.1931	729.05, 687.15, 571.02, 529.05, 409.12, 381.05, 357.06	2
Flavonoids and derivatives	14	Apigenin	C_15_H_10_O_5_	269.0454	269.045	241.01, 151.01, 149.03	1
	15	Luteolin	C_15_H_10_O_6_	285.0405	285.0399	267.05, 241.03, 151.00, 133.02	1
	16	Catechin/Epicatechin	C_15_H_14_O_6_	289.0718	289.0712	245.04, 205.05, 179, 151.04, 137.02	1
	17	Chrysoeriol	C_13_H_16_O_8_	299.0561	299.0555	285.03, 255.02, 153.01, 135.03, 125.03	2
	18	Quercetin	C_15_H_10_O_7_	301.0352	301.0348	273.04, 257.04, 179.00, 151.00	1
	19	Taxifolin	C_15_H_12_O_7_	303.0511	303.0504	285.04, 275.02, 241.05, 151.04, 125.02	2
	20	Epigallocatechin	C_15_H_14_O_7_	305.0637	305.0661	287.05, 137.02, 125.02	1
	21	Methoxysinensetin	C_21_H_22_O_8_	401.1299	401.1236	371.11, 339.08, 191.71	2
	22	Epicatechin hydroxybenzoate	C_22_H_18_O_8_	409.0924	409.0923	289.07, 271.06, 137.02, 119.01	2
	23	Naringenin rhamnoside	C_21_H_22_O_9_	417.1249	417.1186	271.06, 187.03, 151.00, 119.05	2
	24	Epiafzelechin gallate	C_22_H_18_O_9_	425.0877	425.0872	287.05, 273.07, 169.01, 151.00	2
	25	Apigenin hexoside	C_21_H_20_O_10_	431.0989	431.0978	269.04, 241.01, 151.01, 149.03	1
	26	Naringin	C_21_H_22_O_10_	433.1137	433.1134	271.06, 187.03, 151.00, 119.05	1
	27	Epicatechin gallate	C_22_H_18_O_10_	441.0810	441.0821	135, 169, 273, 371, 399, 413, 427	2
	28	Biochanin A glucoside	C_22_H_22_O_10_	445.1199	445.1135	283.06, 268.03, 239.03, 211.04, 132.02	2
	29	Kaempferol hexoside	C_21_H_20_O_11_	447.0929	447.0927	285.04, 241.03, 151.00, 133.02	1
	30	Taxifolin rhamnoside	C_21_H_22_O_11_	449.1089	449.1089	303.05, 285.04, 275.02, 151.04, 125.02	2
	31	Catechin glucoside	C_21_H_24_O_11_	451.1356	451.1240	289.15, 151.10, 137.08, 123.10	2
	32	Epicatechin 3-(3-methylgallate)	C_23_H_20_O_10_	455.1018	455.0978	289.02, 183.05, 124.01	2
	33	Afrormosin glucoside	C_23_H_24_O_10_	459.1354	459.1291	297.07, 281.04, 267.06	2
	34	Chrysoeriol hexoside	C_22_H_22_O_11_	461.1085	461.1083	299.05, 285.03, 153.01, 135.03, 125.03	2
	35	Isoquercitrin	C_21_H_20_O_12_	463.0884	463.0876	301.05, 268.01, 179.02, 151.01	1
	36	Epicatechin glucuronide	C_21_H_22_O_12_	465.1036	465.1033	289.15, 151.10, 137.08, 123.10	2
	37	Epigallocatechin caffeate	C_24_H_20_O_10_	467.0980	467.0978	305.06, 287.05, 179.03, 137.02, 125.02	2
	38	Isorhamnetin glucoside	C_22_H_22_O_12_	477.1035	477.1033	315.05, 300.01, 255.05, 179.05, 151.02	2
	39	Luteone glucoside	C_26_H_28_O_11_	515.1615	515.1553	353.10, 311.05, 297.04	2
	40	Isorhamnetin malonyl hexoside	C_24_H_24_O_13_	519.1141	519.1138	315.05, 300.02, 227.01, 204.04, 177.01	2
	41	Luteolin hexosyl sulfate	C_21_H_20_O_14_S	527.0502	527.0495	447.05, 285.01, 241.06	2
	42	Chrysoeriol hexosyl sulfate	C_22_H_22_O_14_S	541.0658	541.0652	299.05, 284.05, 241.02	2
	43	Isoquercitrin sulfate	C_21_H_20_O_15_S	543.0448	543.0444	463.05, 301.01, 268.01, 179.02, 151.01	2
	44	Procyanidin A2	C_30_H_24_O_12_	575.1195	575.1189	539.09, 449.08, 423.07, 289.07, 285.04, 269.04, 125.02	1
	45	Procyanidin B2	C_30_H_26_O_12_	577.1352	577.1346	451.10, 425.08, 407.07, 289.07, 287.05, 269.04, 125.02	1
	46	Luteolin rhamnosyl hexoside	C_27_H_30_O_15_	593.1509	593.1506	447.09, 285.03, 153.01, 135.04	2
	47	Chrysoeriol rhamnosyl hexoside	C_28_H_32_O_15_	607.1672	607.1663	461.10, 299.05, 284.03, 153.01, 149.05	2
	48	Isorhamnetin rhamnosyl hexoside	C_28_H_32_O_16_	623.1609	623.1612	477.10, 315.05, 299.05, 165.05	2
	49	Isorhamnetin dihexoside	C_28_H_32_O_17_	639.1556	639.1561	447.01, 315.01	2
	50	Procyanidin B2 gallate	C_37_H_30_O_16_	729.1473	729.1455	451.10, 425.08, 407.07, 289.07, 287.05, 169.01, 125.02	2
	51	Kaempferol 3-(3″,6″-di-*p*-coumaroyl galactoside) (Stenopalustroside A)	C_39_H_32_O_15_	739.1679	739.1663	593.12, 575.11, 285.03, 163.03	2
	52	Quercetin 3-*O*-xylosyl-rutinoside	C_32_H_38_O_20_	741.1846	741.1878	609.14, 301.03	2
	53	Epicatechin-(4beta- >8)-epigallocatechin gallate	C_37_H_30_O_17_	745.1395	745.1404	441.08, 303.05, 169.01, 125.02	2
	54	Luteolin rhamnosyl dihexoside	C_33_H_40_O_20_	755.1990	755.2034	709.16, 593.10, 575.05, 285.01	2
	55	Quercetin rhamnosyl dihexoside	C_33_H_40_O_21_	771.1969	771.1983	609.14, 591.05, 301.03, 153.02, 125.00	2
	56	Isorhamnetin rhamnosyl dihexoside	C_34_H_42_O_21_	785.2110	785.2140	623.16, 477.10, 315.05	2
	57	Quercetin 3-sophorotrioside	C_33_H_40_O_22_	787.1909	787.1933	625.10, 463.09, 301.01	2
	58	Quercetin 3-(6′′′′-*p*-coumaryl sophorotrioside) (Pisumflavonoside I)	C_42_H_46_O_24_	933.2302	933.2300	787.19, 625.10, 463.09, 301.01	2
	59	Quercetin 3-(6″-caffeoyl sophorotrioside)	C_42_H_46_O_25_	949.2223	949.2250	787.19, 625.10, 463.09, 301.01	2
Terpenoids	60	8-Hydroxy-(+)-δ-cadinene	C_15_H_24_O	219.175	219.1748	203.14, 201.16, 179.14	2
	61	Valerenic acid	C_15_H_22_O_2_	233.1544	233.1541	219.13, 189.16, 161.13	2
	62	β-Ionyl acetate	C_15_H_24_O_2_	235.1700	235.1698	193.15, 175.14, 149.13	2
	63	Valerenolic acid	C_15_H_22_O_3_	249.1528	249.1490	231.13, 205.15, 187.14, 177.12	2
	64	Phytuberol	C_15_H_24_O_3_	251.1651	251.1647	233.15, 221.15, 193.12	2
	65	Curcolonol	C_15_H_20_O_4_	263.1288	263.1283	245.11, 227.10, 205.08	2
	66	Absindiol	C_15_H_22_O_4_	265.1445	265.1439	247.13, 221.15, 209.11	2
	67	Acoric acid	C_15_H_24_O_4_	267.1602	267.1596	249.14, 223.16, 181.12	2
	68	Phytuberin	C_17_H_26_O_4_	293.1758	293.1752	251.16, 233.15, 221.15, 193.12	2
	69	Trilobinol	C_20_H_28_O_2_	299.2016	299.2011	283.16, 265.15, 257.15	2
	70	Abietadiene-diol	C_20_H_32_O_2_	303.2330	303.2324	287.20, 257.22, 241.19, 215.18	2
	71	Piperochromenoic acid	C_22_H_28_O_3_	339.2000	339.1960	325.18, 295.20, 189.05, 137.02	2
	72	Eucannabinolide	C_22_H_28_O_8_	419.1710	419.1705	389.16, 371.14, 359.14, 347.14	2
	73	β-Amyrenone	C_30_H_48_O	423.3624	423.3626	407.33, 391.30	2
	74	Cichorioside M	C_21_H_32_O_9_	427.1974	427.1968	265.14, 247.13, 221.15, 209.11	2
	75	Cynaroside A	C_21_H_32_O_10_	443.1921	443.1917	281.13, 263.12, 237.14, 193.12	2
	76	Oleanonic acid	C_30_H_46_O_3_	453.3376	453.3368	241, 323, 341, 379	2
Lignans	77	1,2-Di-(syringoyl)-hexoside	C_24_H_28_O_14_	539.1385	539.1401	359.09, 341.08, 197.04, 153.05	2
	78	Citrusin B	C_27_H_36_O_13_	567.2084	567.2077	405.15, 387.14, 358.14, 209.08, 197.08	3
	79	Lyoniresinol glucoside	C_28_H_37_O_13_	581.2236	581.2234	419.17, 265.10, 247.09	3
Carboxylic acid, fatty acids and amino acids	80	Fumaric acid	C_4_H_4_O_4_	115.0026	115.0037	71.01	2
	81	Succinic acid	C_4_H_6_O_4_	117.0183	117.0187	99.00, 73.02	2
	82	Malic acid	C_4_H_6_O_5_	133.0133	133.0142	115.00, 89.02, 71.01	2
	83	Tartaric acid	C_4_H_6_O_6_	148.9235	149.0086	87.05	2
	84	Ribonic acid	C_5_H_10_O_6_	165.0398	165.0418	149.04, 105.01, 87.00, 75.00	2
	85	Citric acid	C_6_H_8_O_7_	191.0191	191.0197	173.00, 129.01, 111.00	2
	86	Homocitric acid	C_7_H_10_O_7_	205.0349	205.0348	161.04. 143.04. 117.05	2
	87	Lauric acid	C_12_H_24_O_2_	199.1698	199.1698	181.16, 165.13, 163.11, 139.11, 135.11	2
	88	Myristic acid	C_14_H_28_O_2_	227.2014	227.2011	209.19, 183.21, 179.18	2
	89	Methylmyristic acid	C_15_H_30_O_2_	241.2171	241.2167	227.20, 209.19, 183.21, 179.18	2
	90	Palmitic acid	C_16_H_32_O_2_	255.2327	255.233	237.23, 211.24, 197.22	2
	91	16-Hydroxypalmitic acid	C_16_H_32_O_3_	271.2279	271.2273	253.12, 237.22. 225.25, 211.24. 195.21	2
	92	α-Linoleic acid	C_18_H_32_O_2_	279.2328	279.233	261.22	2
	93	Oleic acid	C_18_H_34_O_2_	281.2485	281.2486	263.25, 181.21, 127.25	2
	94	Dihydroxy octadecadienoic acid	C_18_H_32_O_4_	311.2226	311.2239	293.22, 275.23	2
	95	Dihydroxy octadecenoic acid	C_18_H_34_O_4_	313.2383	313.2378	295.23, 277.25, 183.32	2
	96	Dihydroxy octadecanoic acid	C_18_H_36_O_4_	315.2538	315.2535	297.23, 279.25,	2
	97	Trihydroxy octadecadienoic acid	C_18_H_32_O_5_	327.2175	327.2171	309.23, 291.25, 273.23	2
	98	Trihydroxy octadecenoic acid	C_18_H_34_O_5_	329.2332	329.2333	311.25, 293.26, 275.23	2
	99	α-Hydroxybehenic acid	C_22_H_44_O_3_	355.3217	355.3212	337.31, 311.33, 293.32, 281.32	2
	100	26-Hydroxyhexacosanoic acid	C_26_H_52_O_3_	411.3842	411.3838	393.37, 381.37, 367.39	2
Others	101	Dihydrojasmonic acid	C_12_H_20_O_3_	211.1335	211.1334	167.14, 111.08, 59.10	2
	102	N-acetyl-α-neuraminic acid	C_11_H_19_NO_9_	308.0986	308.0987	290.09, 219.06, 200.05, 146.08, 128.07	2
	103	1-Deoxynojirimycin hexoside	C_12_H_23_NO_9_	324.1298	324.1295	161.04, 144.06, 143.03, 113.02	2
	104	Icariside D1	C_19_H_28_O_10_	415.1609	415.1604	398.15, 384.14, 250.12	2

EF: elemental formula; OM: observed mass; CM: calculated mass; CL: confidence level.

## Data Availability

Data will be available upon request.

## Supplementary Materials

The following supporting information can be downloaded at: https://www.mdpi.com/article/10.3390/antiox13020238/s1, Figure S1: The ANN models architecture; Figure S2: The contour plot as a function of ethanol concentration, extraction time and temperature at optimum condition; Figure S3: Mushroom tyrosinase inhibition activity of UMS optimized extract; Figure S4: Effect of UMS optimized extract on MNT-1 cell viability; Table S1: List of antibodies used in this study; Table S2: Independent process variables with experimental ranges and levels for ultrasound assistant extraction of UMS [47].
