# Supplementary material for: Optimization, Metabolomic Analysis, Antioxidant Potential and Depigmenting Activity of Polyphenolic Compounds from Unmature Ajwa Date Seeds (Phoenix dactylifera L.) Using Ultrasonic-Assisted Extraction
Source: Antioxidants (Basel). 2024 Feb 15;13(2):238. doi: 10.3390/antiox13020238 (PMC10886343; doi:10.3390/antiox13020238)
Supplement: Supplementary file 1 [file antioxidants-13-00238-s001.zip › antioxidants-2836502-supplementary.pdf]

Supplementary Data

Ultrasonic-assisted extraction of polyphenolic compounds from unripe Ajwa date seeds (*Phoenix dactylifera* L.): Optimization, metabolomic analysis and depigmentation activity

Fanar Alshammari[1], Md Badrul Alam[1,2], Marufa Naznin[3], Sunghwan Kim[3,4], Sang-Han Lee[1,2*]

[1]Department of Food Science and Biotechnology, Graduate School, Kyungpook National University, Daegu 41566, Korea

[2]Food and Bio-Industry Research Institute, Inner Beauty/Antiaging Center, Kyungpook National University, Daegu 41566, Korea

[3]Department of Chemistry, Kyungpook National University, Daegu, 41566, Republic of Korea

[4]Mass Spectrometry Converging Research Center and Green-Nano Materials Research Center, Daegu, 41566, Republic of Korea

*Corresponding author:

*Correspondence to: Dr. Sang-Han Lee; Department of Food Science and Biotechnology, Kyungpook National University, Daegu 41566, Korea, Phone: (82)053-950-7754 (Office); (82)010-2537-7659 (Mobile); Fax: 053-950-6772; Email: sang@knu.ac.kr

1. Experimental

1.1 Anti-tyrosinase activity

The assessment of the anti-tyrosinase inhibitory potential of UMS extracts followed established protocols, as detailed in prior work (Alam et al., 2023). As a benchmark, arbutin (ARB) was employed as a positive control. The percentage inhibition of tyrosinase activity was computed using Eq. 1.

$$(\%\ inhibition) = \left[\left(1 - \frac{Abs_{sample}}{Abs_{control}}\right)\right] \text{x}\ 100 \text{ ---------------------(1)}$$

where $Abs_{control,}$ and Abs_{sample} represent the absorbance of the control and the sample, respectively. Each sample was subjected to this analysis thrice to ensure robust and reliable results.

1.2 Cell culture and cell viability assay

Highly pigmented human melanoma cells (MNT-1), purchased from ATCC (Rock-ville, MD, USA), were kept alive at 37 °C under 5% CO2 in Dulbecco's Modified Eagle Me-dium (DMEM) supplemented with 10% fetal bovine serum (FBS, Hyclone, Utah, UT, USA), streptomycin–penicillin (100 µg/mL each), 10% AIM-V medium, 0.1mM non-essential amino acid mix and 1mM sodium pyruvate (Invitrogen, Waltham, MA, USA). To confirm the cytotoxicity of UMS the 3-(4,5-dimethyl-2-thiazolyl)-2,5-diphenyl-2H-tetrazolium bromide (MTT) assay was used as described in our previous report [1].

Reference

1. Alam, M.B.; Park, N.H.; Song, B.R.; Lee, S.H. Antioxidant Potential-Rich Betel Leaves (*Piper betle* L.) Exert Depigmenting Action by Triggering Autophagy and Downregulating MITF/Tyrosinase In Vitro and In Vivo. *Antioxidants* **2023**, *12*, 374.

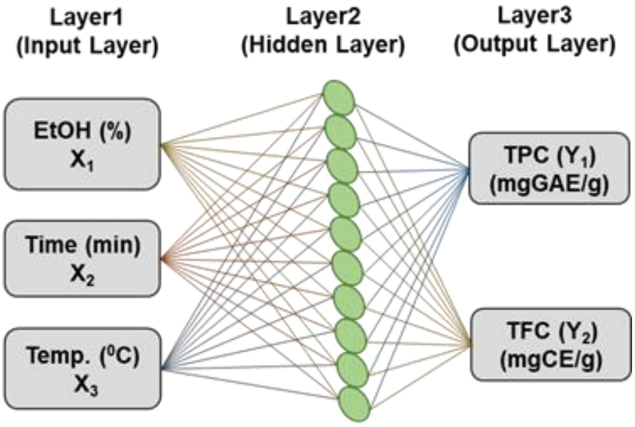

Figure S1. The ANN models architecture.

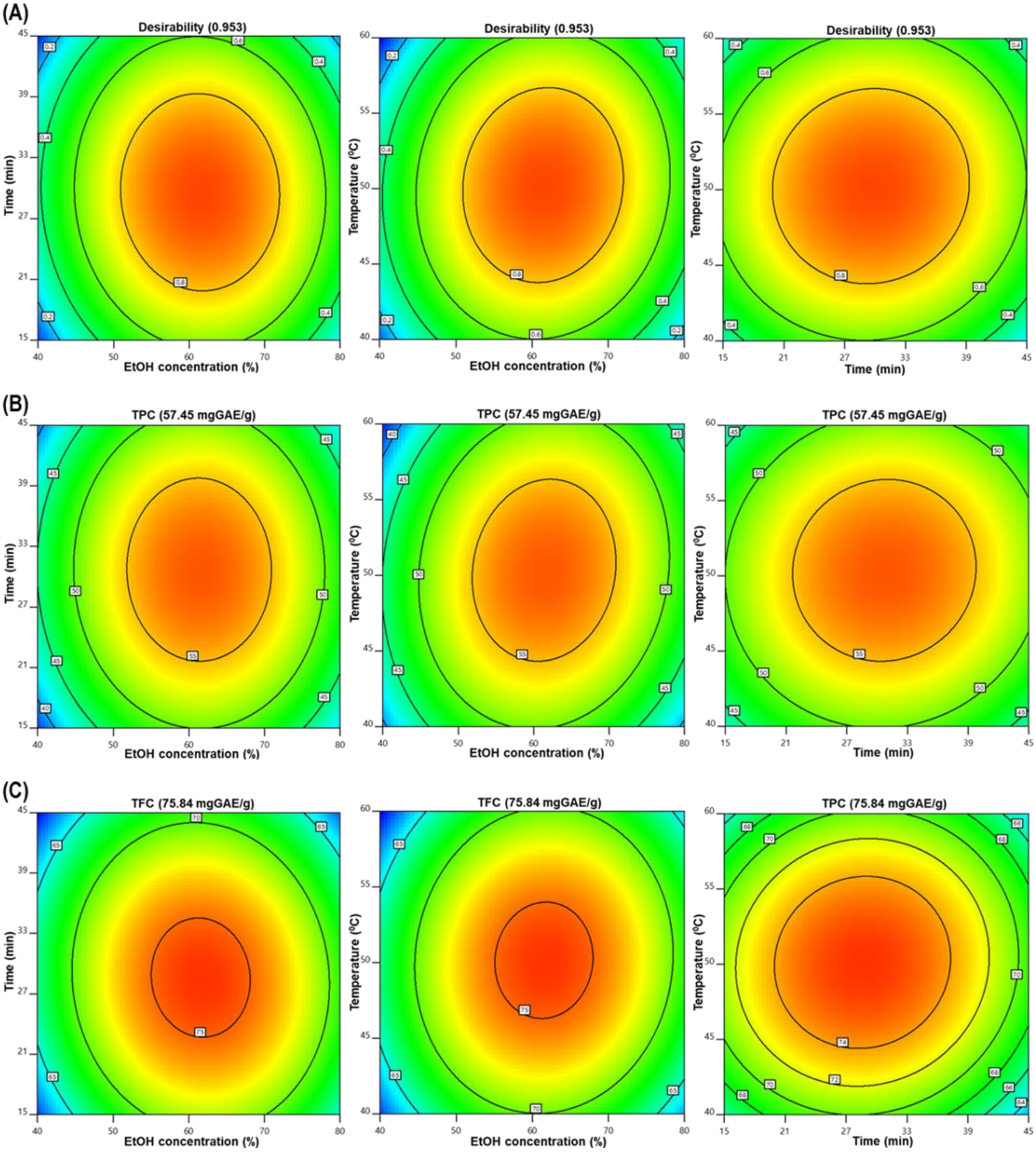

Figure S2. The contour plot as a function of ethanol concentration, extraction time and temperature at optimum condition.

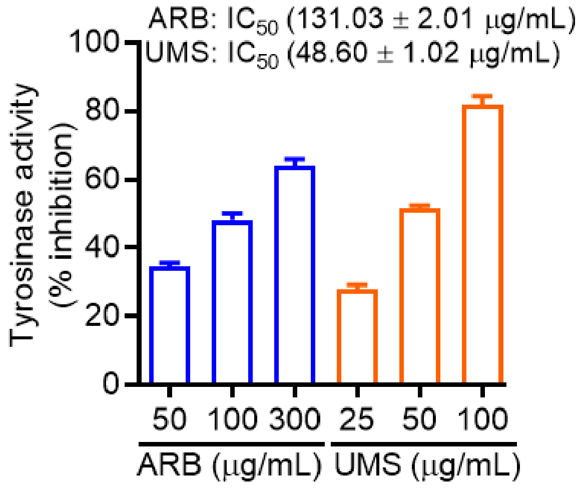

Figure S3. Mushroom tyrosinase inhibition activity of UMS optimized extract. ARB: arbutin.

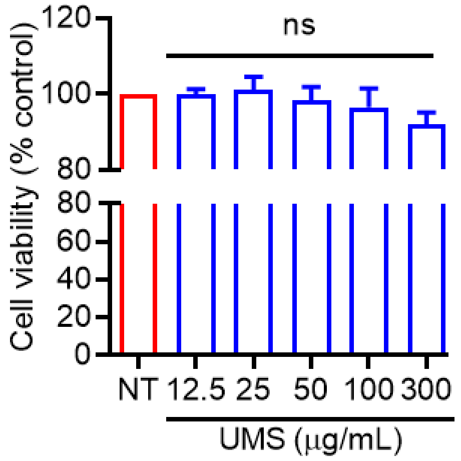

Figure S4. Effect of optimized UMS extract on MNT-1 cell viability.

Table S1. List of antibodies used in this study.

Antibody	Dilution	Supplier	Catalog No.	Host	MW, kDa
Anti-TYR	1:1000	Abcam, Cambridge, UK	ab170905	Rabbit	60
Anti-TRP-1	1:1000	Abcam, Cambridge, UK	ab235447	Rabbit	61
Anti-TRP-2	1:1000	Abcam, Cambridge, UK	ab221144	Rabbit	59
Anti-MITF	1:1000	Abcam, Cambridge, UK	ab140606	Rabbit	58
Anti-p-p38	1:500	Bioworld Technology, Inc.	BS4635	Rabbit	42
Anti-p38	1:500	Bioworld Technology, Inc.	BS3567	Rabbit	42
Anti-p-ERK1/2	1:500	Bioworld Technology, Inc	BS5016	Rabbit	42,44
Anti-ERK1/2	1:500	Bioworld Technology, Inc.	BS 6472	Rabbit	42,44
Anti-JNK	1:500	Bioworld Technology, Inc.	BS3630	Rabbit	46,54
Anti-p-JNK	1:500	Bioworld Technology, Inc.	BS4322	Rabbit	46,54
Anti-β-actin	1:1000	Abcam, Cambridge, UK	ab8227	Rabbit	45

Table S2. Independent process variables with experimental ranges and levels for ultrasound-assisted extraction of UMS.

**Input variables**	**unit**	**Code**	**-1**	**0**	**+1**
**Ethanol concentration**	%	X_1	40	60	80
**Time**	min	X_2	15	30	45
**Temperature**	^0C	X_3	40	50	60
